# Integrating Computational and Experimental Workflows for Accelerated Organic Materials Discovery

**DOI:** 10.1002/adma.202004831

**Published:** 2021-02-09

**Authors:** Rebecca L. Greenaway, Kim E. Jelfs

**Affiliations:** ^1^ Department of Chemistry Imperial College London Molecular Sciences Research Hub White City Campus, Wood Lane London W12 0BZ UK

**Keywords:** automation, high‐throughput screening, materials discovery, prediction

## Abstract

Organic materials find application in a range of areas, including optoelectronics, sensing, encapsulation, molecular separations, and photocatalysis. The discovery of materials is frustratingly slow however, particularly when contrasted to the vast chemical space of possibilities based on the near limitless options for organic molecular precursors. The difficulty in predicting the material assembly, and consequent properties, of any molecule is another significant roadblock to targeted materials design. There has been significant progress in the development of computational approaches to screen large numbers of materials, for both their structure and properties, helping guide synthetic researchers toward promising materials. In particular, artificial intelligence techniques have the potential to make significant impact in many elements of the discovery process. Alongside this, automation and robotics are increasing the scale and speed with which materials synthesis can be realized. Herein, the focus is on demonstrating the power of integrating computational and experimental materials discovery programmes, including both a summary of key situations where approaches can be combined and a series of case studies that demonstrate recent successes.

## Graphical abstract

A discussion of the power of integrating computational and experimental materials discovery programmes for organic material discovery is provided, including the use of artificial intelligence techniques and automation. An overview of the different situations where combined approaches are effective is given, along with a series of case studies demonstrating recent successes.

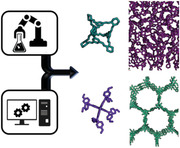

## 1 Introduction

Organic materials have shown potential in a wide range of applications, including gas uptake, molecular separations, as chemical sensors, in catalysis, optoelectronics, and energy storage. There is, however, an ever‐increasing demand for the rapid discovery of new and improved functional materials to address societal challenges such as the capture of greenhouse gases and new catalysts for sustainable living. Arguably, these challenges are ramping up at a rate that exceeds our current ability to address them scientifically. This is because the discovery process is slow, typically taking several years to develop and understand a single new system.

**Figure 1:**
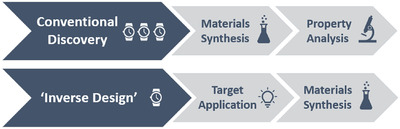
Comparison of conventional materials discovery, where materials are synthesized and then their properties are investigated, to “inverse design” where a material is designed based on a desired target application prior to synthesis. Approximate time cost for each step is indicated (1 clock  =  quick; 3 clocks  =  time‐intensive).

Chemists and material scientists would ultimately like to be able to “design” new materials, with fine control and the ability to tune the properties for specific applications (**Figure** **1**). There are three key hurdles facing us, blocking our ability to do “inverse design,” where we first come up with a wishlist of materials, and then go backward to design a molecule that fits all the necessary criteria. There is no equivalent to the process of retrosynthesis that can be applied to design a route to a desired organic molecular structure. The first challenge for material design comes from the vastness of the search space for potential precursors to organic materials; the number of potential small organic molecules that can be used is mind‐boggling large. Most organic materials are composed of a combination of precursors, creating a further combinatorial explosion in possibilities. It would never be possible to enumerate all these possibilities, let alone synthesize them, so how do we efficiently identify which precursors to use to get new materials with novel functions? The second challenge comes from the fact that it is rarely, if ever, possible to examine an isolated molecule and predict the way in which it will assemble in the solid state. This is a problem because the solid‐state arrangement of molecules influences, if not completely determines, the properties of the materials. The third challenge is that we will always be targeting materials with a desired combination of multiple different properties, and while it may be easy to optimize a single property, that too often comes at the cost of failing to meet criteria for multiple other properties. This task of multiobjective optimization is complex and inevitably a massive hurdle given the intricacy of the relationship between properties in multifunctional materials.

As a result of these challenges, current synthetic discovery programmes can tend to be relatively conservative, with small incremental chemical modifications to known materials typically carried out, where a synthetic chemist can be confident of synthetic success, even if not likely to get large leaps forward in property performance. Alternatively, preexisting known materials are often “reinvestigated” and screened for new applications. Occasionally, serendipity can play a role in unexpected property outcomes or the discovery of a new material class, but this is rare, and we must challenge ourselves to accelerate such events.

Given the vast organic material search space, we would ideally like to synthesize and test extremely large numbers of materials. With automation and robotics, we can hope to increase the number of materials that can be realized and tested, by several orders of magnitude in many cases. However, there are multiple bottlenecks in the experimental screening of materials. Firstly, there is the issue that for every material that is synthesized, the material precursors, small organic molecules, must be either purchased or synthesized. Relying on commercially available precursors can severely limit the available search space, whereas some custom precursors may have challenging multi‐step syntheses, meaning it could take many months to isolate the precursors in sufficient purity and quantity to create a material from it. Precursor synthesis can become a limiting factor when using automation for materials discovery, as the precursor feedstocks need to keep pace with the required throughput. Additionally, significant time investment is typically required in determining the required reaction conditions in order to synthetically realize the material, which can often significantly differ between materials classes. Once synthesized, another significant challenge is the property testing of the materials. Depending on the application and properties sought, this may or may not be suited to any kind of high‐throughput approach. For example, some properties will require specialist, expensive, equipment that can only run samples sequentially rather than in parallel. Many applications, for instance in optoelectronics, may first require time‐consuming device assembly, severely limiting the number of materials that can be tested.

From a computational perspective, we would ideally be able to successfully perform “inverse design,” where we design a molecule that meets a set of criteria for a functional material. However, as outlined above there are significant roadblocks to this capability, originating mostly from the unpredictability of the solid‐state arrangement and hence properties. There is also the danger that computational programmes design a material without consideration of the stability of the system and a viable synthetic route to the material and device assembly. The above type of approach was termed by Jansen and Schön as “putting the cart before the horse,” and they instead put forward that computational materials programmes must focus first on exploring the energy landscape of a material system to find the thermodynamically stable materials, and then screen them for properties to identify the stable, viable, materials that have desired properties.^[^ ^1^ ^]^ In other words, computation cannot be used to “design” materials, but rather to use computational screening to discover them instead.

**Figure 2:**
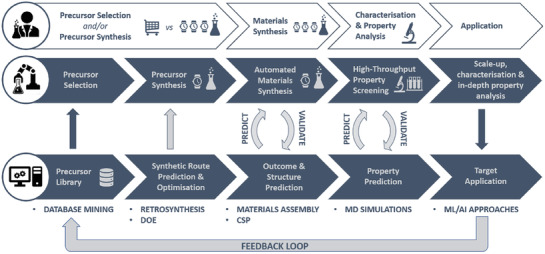
Overall workflow highlighting some of the key discovery steps for new materials, and the key opportunities for combining experiment, whether it be conventional or automated, and computation in a highly cooperative feedback loop to accelerate the discovery process. Approximate time cost for each step is indicated (1 clock  =  quick; 3 clocks  =  time‐intensive).

Herein, we will focus on the potential of integrated materials discovery programmes that leverage the power of combined experimental and computational workflows in a variety of ways. We will outline the various steps to materials discovery (**Figure** **2**), exploring the potential contributions that computation and experiments can play in each of them, which is inevitably dependent on the material class and function. Increasingly, as approaches mature, and as computational predictions become trusted to be more reliable, discovery programmes combine several components, for example, both computational structure prediction and property screening as well as experimental validation. With a series of recent case studies, we will explore examples of how combined computational and experimental programmes have been successful in accelerating organic material discovery, demonstrating progress in various elements of the discovery progress. Finally, we will explore the future outlook for the field, as new approaches including artificial intelligence (AI) and robotics are increasingly applied.

## 2 Steps to Materials Discovery

### 2.1 Material Precursor Selection

The obvious first stage in materials discovery is the selection of the building blocks for the material; in the case of organic materials, the material precursors are (small) organic molecules. Typically, these precursors will require their own multistep syntheses, and most organic materials and devices will include several different organic precursors, and thus the synthesis of the precursors themselves can be a time‐consuming, if not costly, part of the assembly process. Already, this motivates experimental chemists toward a “safe” selection of material precursors, where selected precursors are molecules that can be purchased commercially, ideally cheaply, or molecules with reported syntheses in the literature, or molecules that are close analogues of those previously reported. The unpredictability of material assembly and resultant properties is another barrier; no chemist wants to spend many months synthesizing a costly precursor if the resultant material either does not form or does not have the sought‐after properties. This does however necessarily reduce the chances that a large leap forward in terms of the resultant materials properties can be achieved.

The above limitations are set against a context of a near infinite search space of possible organic material precursors. It is estimated that a combinatorial enumeration of the possible arrangements of organic molecules consisting of 30 or less light atoms reaches ≈10^60^ possibilities.^[^ ^2^ ^]^ This is an incredibly large number, exceeding what could even be enumerated on a computer. Once the fact that most materials and devices would consist of combinations of molecules is considered, the combinatorial explosion in possibilities is, in essence, immeasurable. Here, computational methods have an advantage over synthesis, in that, at least the generation of individual building blocks on a computer is trivial, not the effort of days, weeks, or months. Therefore, if there is the availability to calculate some sort of figure of merit for a molecule, which is effective at identifying promising precursors, many thousands or millions of molecular precursors, dependent on the computational cost of the assessment, can be screened to help identify the most promising molecules, or regions of chemical space, for synthetic targeting. Such computational filtering schemes prior to synthesis are common. As we move toward exascale computing, with massively enhanced computing power speeding up calculations by at least a couple of orders of magnitude, we will be able to screen even larger databases, including at the quantum chemical level, and speed up ML predictions.

While a synthetic chemist may be naturally inclined to make relatively conservative selections of precursors to synthesize, this will typically limit their search to local regions of chemical space, close to precursors similar to those of materials previously synthesized, or previously reported organic molecules. Given the vastness of the chemical space of possibilities, there must undoubtedly be missed opportunities—molecular precursors that lie in different regions of chemical space, that if synthesized would form materials with truly novel properties, for instance by forming a new class of material. While large‐scale computational screening can help us push out into bigger regions of chemical space, methods that enumerate hypothetical molecules based on very small‐scale building block libraries (<100) are not really facilitating greater exploration of the chemical space. Instead however, AI techniques hold some hope in achieving molecular generation by suggesting molecules that truly break out of the local chemical space to generate “wild card” suggestions for materials precursors. Generative AI models have been reported in the field of drug discovery, but little reported in the field of materials, often limited by the lack of availability of sufficiently large databases of molecules with known properties. We have recently demonstrated the application of a recurrent neural network (RNN) and transfer learning to perform molecular generation for donor–acceptor molecules with targeted properties.^[^ ^3^ ^]^ The generative model was able to generate novel donor–acceptor molecules, both rediscovering experimentally known design rules, with atomic substitutions such as halogenation, and via more novel molecular features.

The issue with computational generation of suggested organic molecules as material precursors, whether using AI or not, is that it is highly likely that many structures are not chemically feasible, either due to instability or the fact that there are no viable, or at least known, synthesis routes for their experimental realization. This can be a particular problem when computational discovery programmes are carried out in isolation, without the guidance or insight of experimental chemists—another benefit of integrated computational‐experimental programmes. There are well‐established algorithms that can score organic molecules by their ease of synthesis, for example, the “synthetic accessibility” score developed by Ertl and Schuffenhauerp.^[^ ^4^ ^]^ However, scoring molecules remains challenging, given that when compared to experimental chemists’ rankings, it is common for their to be a large disparity in the rankings of different chemists. Furthermore, these algorithms have not been developed with the synthesis of organic molecules as material precursors in mind. For materials, there are specific requirements, typically that a molecule can be produced at low cost and in a multigram quantity suitable for subsequent material synthesis. One common route to circumvent this is for promising molecules identified by computational screening to then be inspected and selected by experimental chemists for synthesis. However, this does have the problem that it will tend to lead to molecules being selected that are more similar to those previously synthesized, negating the greater potential audacity of computationally generated molecules.

### 2.2 Precursor Synthesis

In order to investigate more of the available chemical space and make more imaginative leaps in discovery, it is important not to have an over‐reliance on commercially available precursors. However, typically the synthesis of custom precursors for use in subsequent materials synthesis is costly and time consuming, especially if multiple steps are required. Depending on whether or not there is a known route to the desired precursor can also exacerbate both of these demands. For example, if a precursor is deemed “synthetically viable” based on analogous compounds, but is novel, a new synthetic route needs to be designed and there is no guarantee this will work first time, meaning multiple alternative routes may need to be investigated. Even when a successful route to the precursor has been realized, it may still take considerable effort and time to synthesize enough material of high enough purity for use in realizing and optimizing the subsequent materials synthesis.

If the synthesis of the precursors involves long and labor‐intensive multi‐step synthetic routes, a number of approaches can be used to minimize the risk while also potentially reducing the timescale in which they are accessed. For example, rather than relying on a high‐risk linear synthesis, where each step is carried out sequentially and therefore the synthesis is reliant on every step working as expected, if possible, a convergent synthetic route could be designed. By splitting the route into two parallel synthetic approaches, the risk is limited, the overall yield potentially improved, and steps can be carried out in parallel reducing the overall time required to access the desired precursor. Alternatively, a divergent approach could be utilized to rapidly access a family of structurally analogous precursors that could be screened for materials synthesis, reducing the risk by not solely relying on a single precursor.

During route development, there might also be a point where new synthetic steps need to be developed, or the reaction conditions of low yielding transformations optimized. Here, automation can potentially be used to streamline and accelerate this process. For example, high‐throughput screening can be used to investigate a large number of experiments in parallel, screening a range of conditions aimed at optimizing a particular transformation, such as different catalysts and solvents. Alternatively, rather than screening every possible permutation of conditions, if design of experiments (DOE) is also incorporated in the optimization of particular reactions, this can aid in reducing the number of reactions that need to be carried out, potentially saving significant time and costs. Another option is to use an optimization algorithm^[^ ^5^ ^]^ in combination with a continuous‐flow platform for the automated optimization of a specific chemical transformation, which can then be expanded to expand the substrate scope.^[^ ^6^ ^]^ Machine learning (ML) can also be used here to aid in the synthesis of small organic molecules,^[^ ^7^ ^]^ for example, by automating the self‐optimization of chemical reactions,^[^ ^8^ ^]^ including those that involve multiple steps,^[^ ^9^ ^]^ and searching for new chemical reactivity.^[^ ^10^ ^]^

So far, these approaches have mostly been applied to small organic molecules that are pharmaceutically relevant, although there is no reason that these techniques cannot be used to synthesize new materials precursors. Additionally, the use of automation and high‐throughput screening to expand the substrate scope and synthesize families of precursors suitable for use in materials synthesis means that it would be possible to produce custom feedstocks that can keep pace with high‐throughput materials screening, reducing the reliance on commercially available precursors. However, this would require robust and process‐friendly routes to be developed for the precursors, alongside methods for rapid purification which often hamstrings high‐throughput approaches.

### 2.3 Forming Desired Products

**Figure 3:**
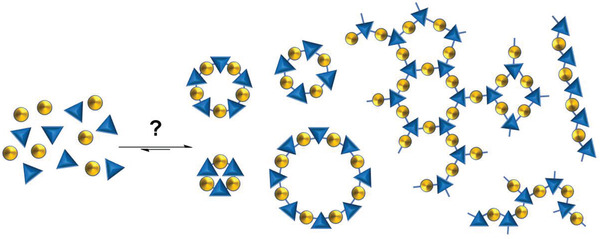
A range of different possible products can be formed from the combination of two organic precursors, including both molecular and extended network structures. Not all possible products are shown.

**Figure 4:**
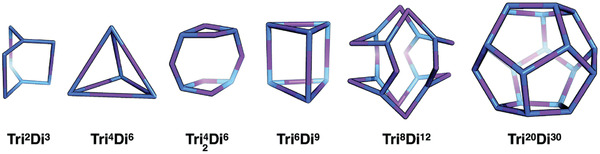
The family of cage topologies that could potentially form from the combination of a tritopic (**Tri**, blue) and ditopic (**Di**, purple) precursor. The superscripts in the labels correspond to the number of each precursor type that is included. Reproduced under the terms of the CC‐BY Creative Commons Attribution 3.0 Unported license (https://creativecommons.org/licenses/by/3.0/).^[^ ^11^ ^]^ Copyright 2017, The Royal Society of Chemistry.

Once precursors are obtained, the next step is to synthesize the actual material. There remain many possible hurdles to a specific desired material being successfully obtained. First, organic precursors can potentially react to form many different products, both molecules of different molecular weights, with different connectivities of the building blocks, or into polymeric, extended structures (**Figure** **3**). For example, in the field of molecular cages, for a given pair of precursors with a specific topicity, there are a whole family of topologies that could form, with different multiples of molecular mass (**Figure** **4**), or a reaction could get “stuck” at an oligomeric intermediate. At the same time, a reaction could be unsuccessful, or form a polymer rather than a discrete product. It is also possible that product mixtures could form or that a desired material could be difficult to isolate or to purify.

Given the large number of possible assemblies for many organic materials, and the vast precursor search space, it is important to be able to automate the assembly of the hypothetical possibilities. We have developed the open‐source *supramolecular toolkit (stk)*, which takes input precursors in a range of formats, including 2D SMILES strings, and assembles them into a range of material classes, including cages, polymers, and frameworks, and predicts their low‐energy conformations.^[^ ^12^ ^]^ Even with the possibility to assemble 100 000s of molecules in minutes, determining which is most promising is too computationally expensive to extend to very large search spaces. We therefore recently extended *stk* to include an evolutionary algorithm, mimicking “survival of the fittest in nature,” to more efficiently sample the chemical space of precursor molecules in order to target materials with specific combinations of properties.^[^ ^13^ ^]^ This software has been successfully applied across a range of porous molecular materials^[^ ^14^, ^15^, ^16^ ^]^ and polymers,^[^ ^17^, ^18^, ^19^ ^]^ with a variety of targeted properties.

In our previous work on the automated synthesis of porous organic cages, out of a set of 78 precursor combinations that were designed by synthetic chemists to be suitable for forming cages, there was a successful “hit” rate of only 42%, with 33 new cages being produced cleanly.^[^ ^20^ ^]^ In this case, a single set of reaction conditions was used, based on a successful setup for one system. One of the “failed reactions” in that study was however found to be successful by Mastalerz et al. on the lab‐bench, using different reaction conditions.^[^ ^21^ ^]^ This demonstrates one reason that a material synthesis reaction can fail—because the correct reaction conditions, such as solvent, concentration, temperature, or reaction length, were not used. Other reasons that you might not form a desired material include that your targeted product is not thermodynamically stable, especially since many organic materials are synthesized with reversible chemical mechanisms, such as dynamic covalent chemistry, where given sufficient time one should stand a reasonable chance of reaching the thermodynamic product. However, there can also be kinetic trapping of an intermediate, or solubility issues, whereby either one of the reagents or one of the reaction intermediates is not soluble and thus the reaction does not proceed to completion.

While automation can obviously assist in finding the optimal experimental conditions under which a targeted product will form, effectively by mapping out phase diagrams for the synthesis of the single material, this is a time‐consuming and costly way to realize a material. It also results in the true potential of automation and robotics not being realized in materials discovery; the robot is just exploring how to optimize the conditions for a single synthesis, potentially carrying out hundreds of experiments to do so. If we could reliably predict the optimal reaction conditions, the robot could instead be testing hundreds of different materials, exploring material not reaction phase space.

We do not generally have the capability to predict the optimal reaction conditions for the synthesis of a targeted product across different material classes. Although, in one case study later, we explore how AI can be used to predict the optimal reaction conditions to optimize the material synthesis and hence properties in metal–organic frameworks. Again, it comes back to the fact that “design” is more challenging than to instead screen for likely assemblies and then select them for synthesis. Here computational prediction can be of more use. For example, porous organic cages, typically synthesized by imine condensation, a reversible reaction, will often form a thermodynamic product. That means that we are able to assemble computer models for each of the possible assemblies, search for their individual low energy conformations and then compare the relative energies of their assemblies, typically using density functional theory (DFT) to get reliable energies.^[^ ^11^ ^]^ These approaches are simplifications, only considering isolated, gas phase molecules, and not the known potential influence of solvent.^[^ ^22^ ^]^ However, there can be considerable success in the reaction outcome prediction, certainly compared to human guesswork, in these systems that can be highly sensitive to small changes. For example, the addition of only a single CH_2_ group to a precursor can double the mass of the resultant cage, and completely change the resultant material properties.^[^ ^23^ ^]^

When the solid‐state material is isolated, it is almost always important that the material is produced and able to be processed into a desired form, as the properties of the material will almost certainly be dependent on the molecular assembly. Computation can assist with the prediction of molecular assembly, as will be discussed in the following section. It is typically possible to control, for example, by choice of synthesis route or drying procedure, whether the material is crystalline or amorphous, although precise control of crystallinity, purity, and defects is more challenging, and again, can heavily influence properties. For example, for organic cages, the porosity of a “crystalline” sample can almost double depending on the drying procedure used.^[^ ^24^ ^]^ Defects, grain boundaries, and the structure of interfaces in devices will also heavily influence properties, for example, charge mobilities in optoelectronic devices.

### 2.4 Structure Prediction

For organic materials, the structure formed in the solid or solution‐state is likely to have a significant influence on the properties or performance of the material. The first element of structure prediction for a molecular material is to correctly predict the molecular conformation(s) adopted. This can be part of the process for predicting the reaction outcome, as discussed above. Molecular prediction should be relatively straightforward, depending on the size and flexibility of the molecular system, as it would typically require finding the global, or low energy conformation(s) of the system. This is a common task in computational chemistry and there are many conformer searching algorithms and approaches that can be applied, either in combination with molecular mechanics if there is an accurate forcefield, or with electronic structure methods. A representative molecular conformation is typically important even for molecular level screening of properties, as it can influence features such as charge mobility, molecular shape, and cavity size.

In the Introduction, we discussed the inherent unpredictability of molecular self‐assembly. This is true for a chemist examining a molecule and predicting the detail of its solid‐state structure. However, there has been much progress in recent decades with the development of crystal structure prediction (CSP) techniques that can take a molecular structure as input and predict the crystal packing.^[^ ^25^ ^]^ These approaches were originally developed for the prediction of pharmaceutical polymorphs, and in blind tests have had increasing success, including effective predictions of large flexible molecules, salts, and hydrates.^[^ ^26^ ^]^ CSP works on the premise that the crystal packing of materials is thermodynamically driven, such that we can expect that experimentally observed polymorphs are either the global minimum polymorph, or a polymorph lying within a few kJ mol^−1^ of the global minimum. Most CSP methods rely upon first conducting a global search for possible polymorphs and ranking their relative energies—at this stage typically 100 000s of packings are tested. A crucial part of the increasing success of CSP has come from the possibility, due to enhanced computational power, of then applying an energetic ranking of low‐energy polymorphs using electronic structure methods, typically DFT calculations with a good description of dispersion forces.

Although the development of CSP methods was driven by the pharmaceutical industry, in the last decade these approaches have begun to be applied in the field of materials science.^[^ ^27^ ^]^ This includes success in predicting the observed crystal structure and preference for enantiopure or racemic packings in porous molecular materials,^[^ ^28^, ^29^ ^]^ which heavily influences the porosity of the materials, as well as the prediction of the packing of organic semiconductors.^[^ ^30^, ^31^ ^]^ These approaches have begun to be integrated with experiment, most notably by Pulido et al., who carried out CSP on a series of small molecules, and the hypothetical polymorphs were then tested for their methane storage capacity to generate “energy–structure–function” maps.^[^ ^32^ ^]^ The identified low density molecular structure was then synthesized, many months of work, and the computational predictions validated. By identifying which of the nine molecules computationally screened had the most promising properties, many years of experimental effort on molecular synthesis and crystallization were not wasted on the less promising systems.

By virtue of the fact that many thousands of polymorphs need to be tested and reliably energetically ranked, CSP is currently computationally expensive. This, combined with the expense of many property calculations, inherently limits the number of molecules that can be “screened” for their crystal packings and accurate solid‐state properties. For example, in the largest scale screen to date, 28 molecules, with ≈20 nonhydrogen atoms in each, were screened for their crystal packing and electronic properties.^[^ ^30^ ^]^ Thus, CSP currently represents a bottleneck in the computational discovery process for materials, and to truly screen materials on a larger scale, needs to be implemented within a tiered strategy that considers much larger numbers of materials at a molecular level before a fine assessment of assembly for a few systems. Integrated directly prior to experimental syntheses, as discussed, it can have great value in selecting the most promising molecules for synthesis from a handful. There is considerable promise in the future for accelerating CSP approaches, particularly via the application of ML models, as shall be discussed later.

Not all materials are molecular, or crystalline, in nature. A lack of long‐range order makes the prediction of a material's structure particularly challenging, although the lack of ability to experimentally determine the structure, for example, through X‐ray diffraction, also makes computationally predicted structural models additionally valuable. We have previously applied computational algorithms that simulate polymerization processes, although do not try to directly emulate them, to generate models of polymeric membranes.^[^ ^33^, ^34^ ^]^ Comparing amorphous polymer models generated from different monomer combinations allows one to rationalize or identify which membrane has pore networks with permeance or selectivity most suited to a particular application, including in membranes for flow batteries or in molecular separations.

Structure prediction is a valuable computational tool, potentially to be combined with other computational approaches as part of a filtering approach. Structure prediction can also be integrated with experiment in a variety of ways, in identifying promising systems, or also, should you have an initial experimental hit on a small number of systems, structure prediction, followed by property prediction can narrow down which of the “hits” it is worth going to the effort and expense of scaling up on the lab‐bench for experimental validation.

### 2.5 Property Prediction

The most substantial application of computation in materials discovery to date is in the area of property prediction, which can almost always be accomplished on a much faster timescale than experimental characterization. Obviously, it is critical for the predictions to be accurate, or even if qualitative, to be sufficient for filtering large numbers of hypothetical materials to the most promising for material synthesis. Experimental characterization of materials is slow, even beyond the time to synthesize precursors and materials, and potential device fabrication, it may be that specialist equipment is required to assess properties such as charge mobility, guest separation, or catalytic activity. Testing each property may be time‐consuming and labor intensive, removing the possibility of high‐throughput property screening, even if the material synthesis is automatable. In some cases however, it may be that while a property cannot be accurately measured in a high‐throughput manner, it is possible to measure a related property, or series of properties, that is indicative of the material's performance. For example, for materials for photocatalytic water‐splitting, high‐throughput characterization (including powder X‐ray diffraction, Fourier‐transform infrared spectroscopy, fluorescence spectroscopy, and time resolved single‐photon counting), and property screening (including porosity) can be carried out, and correlated with the hydrogen evolution rate which can be tested using a high‐throughput photoreactor and gas chromatography.^[^ ^35^ ^]^

The type of calculations applied to property prediction is dependent on the properties or, more commonly, combination of properties being sought, ranging from simple structural assessments to electronic structure calculations. Beyond a single property, a figure of merit from a combination of properties may be targeted, for example, the power conversion efficiency of a bulk heterojunction solar cell material, which depends on the open‐circuit voltage, the short‐circuit current density, and the fill factor. To speed up electronic structure calculations, semiempirical calculations are increasingly used, either calibrated to experimental data, where available, or to higher‐level calculations. Another approach to faster property assessment, which we have frequently used for porous molecular materials, is to assess the individual molecular components of the material, rather than the solid‐state structure, which would take weeks to predict computationally. This “molecular approximation” is possible for many porosity‐related features of porous molecular materials, as these are typically controlled by the molecular structure. For example, automated structural assessment of the windows and cavity size of a porous cage^[^ ^36^ ^]^ can determine bulk performance in encapsulations and separation.^[^ ^37^, ^38^ ^]^ Where molecular simplifications are not possible, calculations on solid‐state properties from hypothetical polymorphs from CSP can be carried out, as done so far for gas storage,^[^ ^32^ ^]^ separation,^[^ ^39^ ^]^ and charge mobility.^[^ ^30^, ^31^ ^]^

AI is well suited to the types of forward predictions that are involved in making property predictions based on training supervised ML models, or exploring structure–property relationships with unsupervised ML. Supervised ML allows property predictions to be made in the order of seconds for a system, rather than minutes, hours, or days. Examples of the application of supervised ML for rapid property prediction span the range of organic materials, including random forest models for prediction of porosity in 66 000 porous organic cages^[^ ^15^ ^]^ and neural networks for optoelectronic properties, such as ionization potential, electron affinity, and optical gap.^[^ ^19^ ^]^ There are a couple of key requirements for successful ML predictions, first, training data for the model is required in sufficient quantity and quality. The exact quantity required is dependent on the type of model being applied, with regression and decision‐tree models having lower requirements than neural networks. Second, a descriptor for the material is required, to represent the key features of the model in a numeric, often vector‐based, representation.

With regards to databases containing properties, these are comparably sparse in the field of organic materials, certainly compared to inorganic materials, where there are large‐scale databases such as NOMAD^[^ ^40^ ^]^ and the Materials Project.^[^ ^41^ ^]^ One difficulty is the lack of a uniform way to represent the structures of organic materials and potentially complex device architectures, and the fact that large numbers of materials have not been synthesized and characterized, certainly not in a consistent fashion suitable for training data. Specific databases with experimental data often only contain a few hundred data points, thus the majority of organic material databases instead contain computed data, where there are 10 000s of data points for a specific application, for example, the electronic band structures in the Organic Materials Database.^[^ ^42^ ^]^ This means computational studies that aim to use ML to predict properties typically have to start by building their own training data first, taking care to have a diversity of systems such that the scope of the model is as broad as possible. With regards to the representations of the materials, these need to capture key chemical features of the material, which will often include solid‐state structural arrangements. The representation required will be dependent on the properties being predicted, in some cases a 2D graph representation will be sufficient, but this is unlikely in the case of electronic properties that depend on molecular conformation, or even crystal packing. The development of improved descriptors for molecular packing is required for many systems to be adequately modeled by ML, for example, the SOAP kernel includes how similar the 3D arrangement of a system is.^[^ ^43^ ^]^ Deep learning (DL) algorithms can generate their new representations of structures through automated feature extraction.^[^ ^44^ ^]^ DL has thus far been mostly applied for the prediction of molecular properties, such as energies, but is likely to see increasing use in organic materials.

Computational property prediction is extremely valuable at narrowing down large search spaces for organic materials, to target experimental testing upon the most promising regions, especially when these are materials that would not otherwise have been considered or designed based on intuition alone. This allows automation to be focused upon a smaller range of possibilities.

### 2.6 Feedback Loops and Experimental Input into Computation

Integrated feedback loops in the materials discovery process can help to streamline and accelerate the overall process. In particular, this can aid in refining the computational models, and trends discovered during experimental screening of materials synthesis and properties can feedback into the selection of new precursors. For example, if there are families of particular precursors that do not successfully form the desired organic materials, even after screening a wide variety of reaction conditions, these can be removed from any subsequent iterations of the workflow.

Experimental insight can provide guidance into which systems are synthesizable, allowing selection of materials, but, further, experimental data are incredibly important both for validation of predictions, and revisiting of approaches, and to build databases of materials and their properties for training by ML models. Here, automation and robotics provides the potential to vastly increase the scale, and decrease the timescale, with which data are collected. Integrated programmes can best utilize this opportunity, ensuring that data are collated and archived in a consistent fashion, including the recording of the system, the synthesis conditions, and any known details of the structure and properties. Very often, this information is not recorded electronically and certainly not made open‐source. Computational researchers can provide guidance on the scope and diversity of systems they would like to be experimentally characterized, emphasizing the need for the inclusion of failed or unsuccessful experiments as well, which may well be the majority of attempts in materials chemistry. Large open‐source libraries of material properties hold enormous promise for the development of more ML models for rapid property prediction, vastly increasing the scale of systems that can be computationally screened.

Another potential source of materials data that is effectively unharnessed at present is the body of historic chemical literature. A combination of supervised and unsupervised ML can be used to mine both the text and images of reports of material synthesis and characterizations, to create databases of known materials and their properties. One example of this, which is discussed further in the case studies, is the chemical data extractor (CDE),^[^ ^45^ ^]^ which makes use of natural language processing and named entity recognition, and has already been used in the field of materials science for discovering organic dyes for solar cells in a combined computational‐experimental study.^[^ ^46^ ^]^ Recently, Terayama et al. demonstrated the use of ML to search for promising optical properties for potential materials application in a preexisting database of drug candidates, finding several promising candidates.^[^ ^47^ ^]^ Not only is this an example of mining known structures, it also demonstrates an example of missed opportunities when systems are only considered for one application and never screened for other functions.

Material databases can be explored to construct and understand structure–property relationships, to assist in the development of design rules. With material compositions in a database, and more ML models for property prediction, materials can be revisited for completely different properties to those which they were originally intended or characterized for. For example, a database of porous molecular materials could be computationally screened for optical or magnetic properties and promising materials identified for synthesis. This could help deal with the undoubtable “missed opportunities,” where a material is only ever tested for a single function, and so some alternative “wonder” property that it possesses might not be discovered even when the material itself is in‐hand.

## 3 Case Studies

We will now discuss a set of case studies that highlight the value of combined computational and experimental workflows to accelerate the discovery of functional organic materials. These case studies are not selected to be exhaustive, but rather to exemplify recent successes that have been applied to different components of the above outlined discovery steps for materials discovery.

### 3.1 Computationally Driven Discovery of a Porous Organic Cage Using a Nonintuitive Precursor

Computation can be used to guide the discovery of porous organic cages,^[^ ^11^, ^48^, ^49^ ^]^ by predicting the molecular topology that is likely to form and whether it will be shape persistent or collapse. Typically, these studies have focused on a small number of molecules, using commercially available or simple precursors provided by experimental researchers, and have often been used for a posteriori rationalization. However, Berardo et al. used the “*Supramolecular Toolkit* *(STK)*”^[^ ^12^ ^]^ to automate the computational assembly and screening of 10 000 possible combinations of precursors, sourced from the Reaxys database, into organic cages, with the aim of discovering promising and synthetically viable precursors.^[^ ^14^ ^]^ While the vast majority of combinations formed cages that lacked shape persistence, a promising tritopic precursor was identified that was predicted to form a number of shape‐persistent cages. Perhaps most importantly, by using a database of molecules that are known to be synthetically viable, and therefore removing the bias based on chemical intuition on what makes a good cage precursor, in this case that the building block should have the same degree of symmetry relative to its topicity, a precursor that would not have been designed or selected based on existing chemical knowledge was selected for experimental investigation.

**Figure 5:**
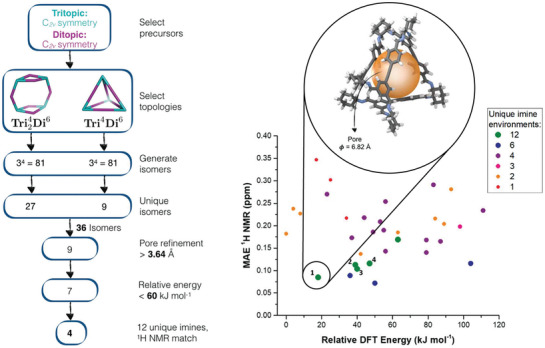
(Left) Computational pipeline for generating and screening potential structural isomers, which in combination with experimental data filtered down to four plausible candidate isomers. (Right) Comparison of the 36 unique isomers taking into account the relative DFT energy, the number of unique imine environments, and the mean absolute error (MAE) between the experimental and calculated ^1^H NMR spectra. The final four candidate isomers are labeled, with the structure of the proposed isomer having the lowest relative energy and the lowest MAE of the ^1^H NMR shifts, shown. Reproduced with permission.^[^ ^14^ ^]^ Copyright 2018, The Royal Society of Chemistry.

Inspired by the computational identification of a tritopic precursor with C_2v_ symmetry, a high‐throughput automated screen was then carried out with a range of linkers and the formation of a **Tri^4^Di^6^** cage species that lacked any symmetry elements was discovered, which was both porous and highly soluble. Due to the use of the tritopic C_2v_ precursor, and the formed cage stoichiometry, the cage could have been one of 162 different possible structural isomers, and experimental analysis techniques alone could not identify the cage isomer. Therefore, computation was used for the challenging task of narrowing down the possible structural isomers to the most plausible candidate. A combination of DFT calculations to determine the relative stabilities of the isomers, pore size calculation, and consideration of the number of unique imine environments (i.e., only isomers with 12 unique imine environments were carried forward), was used to reduce the number plausible candidates to 4 (**Figure** **5**). For this reduced number of structural isomers, NMR shifts were then calculated, which when combined with the experimental data, led to a single isomer being proposed as the most plausible structure.

Overall, this combined computational and experimental study removed selection bias based on prior chemical knowledge and inspired the choice of precursor, therefore enabling the discovery of an unsymmetrical organic cage which would not have been discovered based on intuition alone. In addition, Berardo et al. also studied the origin of porosity and solid‐state structure of the amorphous solid, which is not possible by experiment alone, further highlighting how combined computational‐experimental programmes can provide additional insight into organic materials.

### 3.2 Combined AI Route Prediction with Robotics for Small Organic Molecule Synthesis

The ability to make the entire process, from design through to synthetic realization of a target organic molecule, autonomous, would be a new paradigm for chemical synthesis. Coley et al. took a major step toward this ambitious aim by using AI to automate both the retrosynthetic analysis and planning of an experimentally viable forward synthetic route of an organic molecule, which was then executed using robotics.^[^ ^50^ ^]^

The integration of computer‐aided synthesis planning (CASP) with automated chemical synthesis is challenging, not least because it is difficult to predict specific and feasible reaction conditions for a forward reaction without human intuition, and the range of chemical reactions that can be carried out on a specifically configured automated platform can be limited. In order to overcome these hurdles, Coley et al. developed open‐source CASP software, trained using Reaxys and patents, that could retrosynthetically analyses compounds, identify forward reaction conditions, and also evaluate the likelihood of success. These synthetic routes were then carried out using a modular robotic flow platform that is reconfigurable, providing the flexibility required for different reactions. Unlike most conventional automated platforms that are configured manually by the user, the required configuration was setup using a robotic arm. However, the AI proposed synthetic routes still require input from an experienced chemist to define certain reaction parameters, such as the residence times and concentration, to ensure the process is “flow‐friendly.” Overall, the synthesis of a range of different small organic molecules was successfully predicted and automated using this combined workflow, and while known routes were available for all of the target molecules, the software was prevented from simply selecting these so that all routes had to be discovered based on the learnt transformations and patterns of chemical reactivities.

Although the small organic molecules targeted were predominantly pharmaceutically relevant molecules, and “human‐refined chemical recipe files,” which included adjustments to the AI‐proposed route where required, this study successfully demonstrated that is possible to go from the target molecule to an AI planned route through to automated synthesis, and is a significant milestone toward fully autonomous synthesis. While this approach has not yet been applied to materials precursors, given the potential opportunities for investigating more novel organic materials space and making more drastic leaps in materials properties by using custom precursors, the use of AI to plan a viable synthetic route to a target precursor, combined with robotics to execute said route, would mean that materials chemists, who do not always have a background in organic synthesis, have the opportunity and capability to access novel building blocks, revolutionizing the field.

### 3.3 Computational Outcome Prediction and Robotic Synthesis of Organic Cages

**Figure 6:**
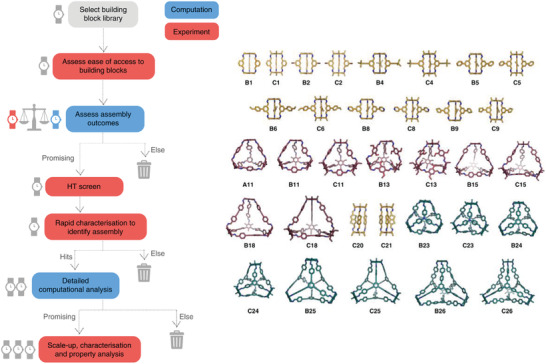
(Left) Hybrid workflow fusing high‐throughput automated synthesis with computation for the accelerated discovery of supramolecular materials. Experimental stages are shown in red and computational stages are shown in blue. (Right) Examples of the different organic cages discovered using computational screening fused with robotic synthesis. Capsular cages incorporating five precursors shown in yellow, tetrahedral cages incorporating ten precursors shown in maroon, and tetrapods incorporating eight precursors shown in teal. Reproduced under the terms of the CC‐BY Creative Commons Attribution 4.0 International license (https://creativecommons.org/licenses/by/4.0).^[^ ^20^ ^]^ Copyright 2018, The Authors, published by Springer Nature.

As discussed above, the topologies and shape‐persistence of porous organic cages can be investigated using computational modeling. However, the extent to which the outcome can be reliably predicted was investigated recently by Greenaway et al., who screened a broad array of 78 different precursor combinations using computation prior to investigating the synthetic outcome using an automated platform.^[^ ^20^ ^]^ Uniquely, this study presented both the limitations of the predictive strategy as well as its successes, and included all of the reactions outcomes, even those that did not lead to the targeted assembly or led to no cage being formed. This resulted in a hybrid computational‐experimental workflow being proposed to streamline the discovery of supramolecular assemblies (**Figure** **6**).

First, computational modeling was used to investigate the topological preferences for each representative family of precursor combinations—for two of the families, the topological preference was more clear‐cut based on comparison of the relative energies, but all three were experimentally confirmed. Then the formation energies were calculated for all of the precursor combinations based on these topology preferences, and their synthesis attempted using high‐throughput automation, before the results from each were compared.

Overall, the success rate from the experimental screen was 42%, but it was clear that it was not possible to unambiguously predict the outcome of each precursor combination, or indeed predict the likelihood that a cage would form. However, comparison of the calculations with the experimental outcomes showed that in future iterations and design cycles, computation could be used to focus experimental efforts. For example, comparison of the formation energies clearly indicated that one particular building block was less likely to be favored in the cage formations, which was then confirmed experimentally, and the most failed reactions occurred in the family where the topological preference was less clear‐cut. Therefore, this approach could be used in future integrated studies to narrow down the search space by ruling out costly and time‐consuming syntheses which are less likely to work, and perhaps more importantly, coupled over many cycles could yield a large database of both successful and failed reactions that could fuel machine learning approaches in the future.

Finally, this study also led to the serendipitous discovery of a new cage topology which would not have been computationally predicted—a doubly bridged triply interlocked catenane, but its formation could be rationalized with it being found to be more stable than the initially formed hit. However, developing a combined computational‐experimental strategy that does not miss serendipitous discoveries, and instead accelerates them, is nontrivial.

### 3.4 Data‐Driven Prediction of Optimal Material Synthesis Conditions

In recent work by Moosavi et al., experimental data on successful and failed syntheses of metal–organic frameworks (MOFs) were collected and then used to train an ML model to predict the optimal synthesis conditions to maximize surface area in the materials.^[^ ^51^ ^]^ Although MOFs are obviously not wholly organic materials, this is an interesting case of modeling being used to guide and accelerate successful synthesis of materials to a desired outcome, that would otherwise rely on trial‐and‐error or an experimental chemist's “chemical intuition” from years of experience in MOF synthesis. MOF synthesis involves the self‐assembly of metal and organic components into a 3D periodic network, and features such as the crystallinity, degree of defects, and consequentially the properties, such as surface area and gas uptake, are known to be dependent on the synthesis conditions for a given system. The search space in a MOF synthesis includes the chemical composition, precursor choice, temperature, solvent, and reaction time. When a new MOF is being synthesized for the first time, this would therefore require between dozens and thousands of experiments to adequately sample the reaction phase space to be confident that the optimal synthesis conditions had been discovered. This is costly and time‐consuming, even with the application of automated synthesis.

The first challenge to trying to apply a data‐driven approach to MOF synthesis is the absence of abundant data on the effect of different synthesis conditions on the reaction outcome. In particular, while successful reaction conditions are reported in the scientific literature, failed experiments, or those that resulted in the material but with suboptimal properties, are not reported. This absence of the “failed data” is a common issue in chemical problems, where indeed the successful, desired, event is likely to be the exception to the experimental attempts, but is the only event typically recorded in the literature. We can hope that an increase in uptake of electronic laboratory notebooks can help with this missing data in the future. To fill this gap in the data, Moosavi et al. used robotic synthesis to collect data on 120 different syntheses of a Cu‐based MOF, HKUST‐1, which can have a Brunauer–Emmett–Teller (BET) surface area ranging from 300 to 2000 m^2^ g^−1^ depending on the solvent composition, reaction temperature, and synthesis method, despite all powder diffraction patterns appearing identical.^[^ ^51^ ^]^

With this synthetic data in hand, Moosavi et al. then used a random decision forest method to determine the relative impact of the different experimental conditions on the synthesis outcome. This uncovered that temperature changes were three times as important as the reactant composition on influencing crystallinity, providing insight that could be applied to future searches. This learning was taken forward to the synthesis of a related Zn‐based MOF, and only 20 samples of the search space were required to find optimal synthesis conditions, compared to an expectation of thousands of samples without the prior insight. Future application of this approach relies upon the availability of databases where successful and failed material syntheses are reported in a common format. Driving uptake of such databases and data‐sharing by the broader community is a significant hurdle to be overcome.

### 3.5 Outcome and Structure Prediction of Multicomponent Self‐Sorted Assemblies

**Figure 7:**
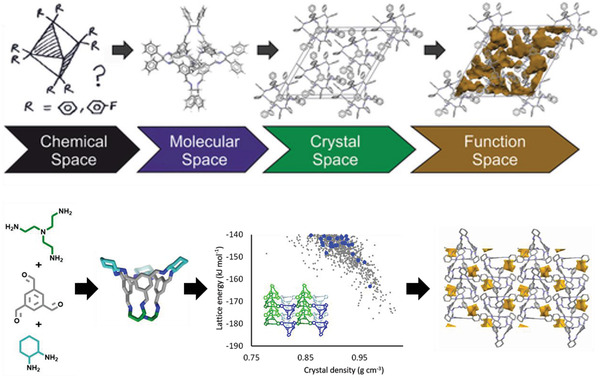
(Top) Proposed general scheme for the de novo structure and property prediction of functional materials by Day and Cooper.^[^ ^27^ ^]^ (Bottom) Outcome prediction of self‐assembled organic cage pot using three precursor building blocks, followed by structure prediction of the crystal packing in the solid state (CSP energy landscape shown for the homochiral derivative, with window‐to‐window packed structures shown in blue and inset of the lowest energy predicted structure), and the available pore space in the global minimum structure. Top: Reproduced with permission.^[^ ^27^ ^]^ Copyright 2017, Wiley‐VCH. Bottom: Adapted with permission.^[^ ^52^ ^]^ Copyright 2019, Wiley‐VCH.

The ability to go from design and precursor selection all the way through to the molecular assembly, solid‐state structure and property prediction, of molecular organic materials, prior to experimental realization, would enable integrated workflows for the in silico design of materials to be realized (**Figure** **7**). Unlike the case studies on organic cages already discussed, which used computational predictions for different stages in the process separately, for example, to identify promising precursors or to predict the most likely molecular outcome from a range of possible topologies, Greenaway et al. recently reported the formation of a multicomponent self‐sorted organic assembly which accomplished this complete workflow.^[^ ^52^ ^]^ That is, the assembly was predicted from the building blocks through to the solid‐state assembly, before being experimentally realized.

First, the molecular space was predicted, which was made more challenging by the use of three building blocks—while the use of two precursors can lead to different topological outcomes, the use of three precursors can also lead to different types of self‐sorting. Computational predictions and comparison of the DFT calculated formation energies for a range of precursor combinations suggested that the formation of socially self‐sorted three‐component organic cage pots was synthetically viable, although the self‐sorted two‐component organic cages were similar in energy. Therefore, based on this prediction alone, it was unclear what species would form experimentally. However, taking into account the entropic contributions to the free energy difference, it was found that certain socially self‐sorted organic cage pots were thermodynamically favored, with the entropic advantage outweighing the formation energy difference. This was then experimentally confirmed using a high‐throughput synthetic screen and an organic cage pot, a new topology in the family of imine‐derived organic cages, was isolated confirming the molecular prediction.

CSP was then carried out to predict the low‐energy solid‐state packing, and while no single structure was dominant, suggesting the system could be highly polymorphic, it indicated that chiral recognition could be expected and that window‐to‐window packings were less preferred in the homochiral structures. Subsequent void analysis to probe the porosity of the crystal structure landscapes suggested that most of the structures would not contain any interconnected pore structures. However, the crystal structures were still investigated experimentally in an attempt to validate the conclusions from the CSP. Unfortunately, only solvated crystal structures were obtained preventing a direct comparison to the predicted landscape. While several of the overall predictions were validated by experiment, such as the crystal structure confirming the molecular assembly and the formation of racemic cocrystals confirming chiral recognition, this highlights that even when the entire workflow can be predicted, there can still be difficulties in experimentally realizing the systems to fully validate the computational results.

### 3.6 Hybrid Experimental‐Computational Approach to Discover Hidden Polymorphs

For organic materials, an ultimate goal is to have the ability to control the solid‐state arrangement of the organic molecules, to allow design of properties. However, crystal engineering remains an ongoing challenge due to the unpredictability of the assembly of a molecule from its molecular structure alone. Cui et al. used a fusion of CSP and high‐throughput crystallization screening to discover new, lower density, polymorphs for two organic molecules that had already been studied over multiple decades.^[^ ^53^ ^]^ Organic molecules that pack with low‐density, open pore structures are a rare exception, as typically molecules will pack efficiently so as to minimize open void space. Thus, the ability to predict the solid‐state structure of these types of molecules to identify those with potential low density packings through computation is powerful.

Trimesic acid was first reported in the solid‐state in 1969 by Duchamp and Marsh,^[^ ^54^ ^]^ with several further crystal structures being reported in the following decades. The CSP study conducted by Cui et al. revealed, however, that there were multiple lower density hypothetical structures with hexagonal hydrogen bonded sheets containing hexagonal pores that should potentially be accessible if supported by the right solvent or solvent combination. This prediction motivated a high‐throughput screen of more than 280 solvent combinations, of which only 6% gave a crystalline structure. Just six new phases of trimesic acid were found, including the predicted one, highlighting the value of the computational screen motivating a search for such rare, “hidden” polymorphs, in a system that had already been extensively studied.

**Figure 8:**
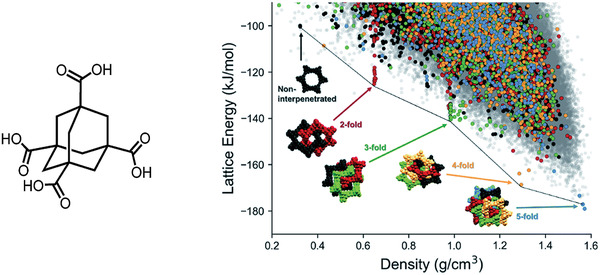
CSP map for ADTA. The different colors highlight the number of unique hydrogen‐bond networks interpenetrated within each structure: blue are fivefold, yellow are fourfold, green are threefold, red are twofold interpenetrated. Charcoal are non‐interpenetrated diamondoid hydrogen bonded networks and light gray dots show structures that do not contain the diamondoid hydrogen bonding. Reproduced with permission.^[^ ^53^ ^]^ Copyright 2019, The Royal Society of Chemistry.

Building on their success with trimesic acid, Cui et al. carried out CSP for adamantane‐1,3,5,7‐tetracarboxylic acid (ADTA), which had previously only been synthesized in a dense fivefold interpenetrated structure. Again, the CSP identified lower density structures that were low‐energy spikes in the energy‐density landscape of hypothetical polymorphs, although higher energy than the previously reported structure (**Figure** **8**). These lower density structures had lower‐fold interpenetration, including one non‐interpenetrated diamondoid structure. This CSP motivated a high‐throughput crystallization screen, which uncovered the conditions for the synthesis of lower‐fold interpenetrated ADTA structures. This study highlights the value of hybrid studies combining computational screening via structure prediction with high‐throughput experimental searches. The CSP had motivated the experimental search, a clear advantage over “blind screening” of systems, and in this case discovering hidden systems that had not been found despite multiple decades of research on those systems. There is thus the potential for such hybrid workflows to accelerate the discovery of organic materials with targeted properties, even beyond simple structural features such as open‐pore frameworks, such as optoelectronic performance.

### 3.7 Navigating the Structure–Property Space of Organic Polymer Photocatalysts

One potential application of organic materials is as photocatalysts for generating hydrogen sustainably through water‐splitting. The photocatalyst needs to absorb light and generate charge carriers that can reduce protons to hydrogen and oxidize water, potentially achieving the latter through a sacrificial donor. While the majority of materials studied as potential photocatalysts are inorganic, some organic materials have been shown to have potential for water‐splitting, or at least for hydrogen evolution, including carbon nitride.^[^ ^55^ ^]^ Since then, a range of organic photocatalysts, including conjugated microporous polymers (CMPs), covalent triazine‐based frameworks (CTFs), and covalent–organic frameworks (COFs) have been reported as organic photocatalysts.^[^ ^56^ ^]^ One potential advantage of organic materials as photocatalysts for water‐splitting is their potential for tunability of properties given the vast potential diversity of the building blocks for these materials. In this context however, only a tiny fraction of the potential organic photocatalysts has ever been tested.

**Figure 9:**
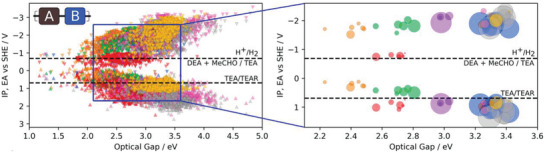
(Left) Predicted optoelectronic properties (ionization potential (IP), electron affinity (EA), and optical gap) of the entire copolymer library (6354 copolymers) of Bai et al.^[^ ^35^ ^]^; (right) Equivalent plot where marker size is proportional to the experimentally observed hydrogen evolution rate, measured for a synthesizable subset of 43 copolymers obtained by combining six dibromide compounds. Different colored points represent systems with different building blocks. Reproduced with permission.^[^ ^35^ ^]^ Copyright 2019, American Chemical Society. (https://pubs.acs.org/doi/10.1021/jacs.9b03591; further permissions related to the material excerpted should be directed to the ACS)

In 2019, Bai et al. reported a tiered strategy involving first computation and then automation to study a much larger range of potential conjugated polymer photocatalysts, likely studying a larger set of materials than previous studies combined.^[^ ^35^ ^]^ They considered a library of commercially available dibrominated arene building blocks that could be combined with diboronic acids or esters via Suzuki–Miyuara couplings to form copolymers. The search space included 6354 candidates, which were first screened computationally using automated assembly of the materials, followed by calibrated semiempirical tight‐binding calculations that provided similar accuracy to DFT calculations, but at a fraction of the cost (**Figure** **9**). The calculations identified a promising subset of 126 copolymers that were selected for synthesis. The synthesis was carried out using a robotic platform for weighing and loading a microwave reactor with the monomers and reagents, followed by testing for hydrogen evolution on a high‐throughput photoreactor. Several new polymers with high sacrificial hydrogen evolution rates were identified.

This tiered strategy, on a larger scale than previous studies, also produced the data to drill down into the structure–property relationships of this class of organic photocatalysts. No single property was found to correlate with performance, and so a gradient‐boosting machine learning model was built from the collected data. This model was able to capture 68% of the variation in hydrogen evolution rate in the conjugated polymers from four descriptors; the computed electron affinity, computed ionization potential, the computed optical gap, and the experimentally determined transmittance values. This was a considerably better performance than from looking at single factors alone; this and the fact that only 68% of the variance was captured demonstrate how clearly many independent factors influence the photocatalytic performance of organic materials. Overall, the study of Bai et al. demonstrates several points; first, the power of combined high‐throughput computational and experimental automated synthesis, beyond what could be achieved by either approach alone given the vast search space of organic materials. Second, the potential for computational screening to narrow down search spaces to promising regions, pushing research into untested areas and finally, that automation and robotics can assist in the general of the larger datasets that are required for building accelerated data‐driven predictions in the future.

### 3.8 Large‐Scale Computational‐Experimental Discovery of Organic Light‐Emitting Diodes (OLEDs)

OLEDs are devices that are made up of molecules that emit light under an applied electric current, and they have application in display units. While there are advantages in synthetic diversity and properties such as flexibility, there are issues with OLEDs requiring higher efficiency, stability, and lower cost. One approach to reduce the cost of phosphorescent OLEDs, which typically rely on low‐abundancy iridium, is thermally activated delayed fluorescence (TADF), where non‐emissive triplet states are harvested via thermal fluctuations that repopulate the emissive single state. In a collaborative study by Gómez‐Bombarelli et al., a large‐scale search for blue TADF emitters for OLED devices was carried out, integrating quantum chemistry calculations, machine learning, synthesis, and device fabrication and testing.^[^ ^57^ ^]^

First, Gómez‐Bombarelli et al. needed to construct a large library of potential OLED candidates. An OLED with TADF needs both donor and acceptor moieties, and so DFT calculations were carried out on a small fragment library to classify them as donors or acceptors. This identified 110 donors, 105 acceptors, and 7 bridges. A combinatorial enumeration algorithm was then used to combine those fragments into all possible candidate OLED molecules, with the synthetic accessibility checked by the algorithm of Ertl and Schuffenhauer,^[^ ^4^ ^]^ to ensure reasonable feasibility of the organic molecules. This resulted in a large library of 1.6 million candidate TADF molecules. Experimentally calibrated quantum chemical calculations were used to assess key properties of the organic molecules, for example, time‐dependent DFT (TDDFT) for emission color. A single figure of merit, an upper bound on the delayed fluorescence rate constant, was used to assess the TADF character of the molecules. However, quantum chemical calculations are too computationally expensive to apply to a library of 1.6 million molecules, and thus instead a machine learning model was built to allow prescreening of the molecules. Using data from previous studies and chemical fingerprints of the molecules, a neural network was used to predict the performance of the molecules. Highly ranked molecules were then run in TDDFT calculations, with continual retraining of the neural network.

900 particularly promising TADF molecules were identified from the 1.6 million candidates, emphasizing the relative rarity of the properties required and the value of high‐throughput computational screening for properties. The identified subset of molecules also included well‐known TADF‐emitters, a promising sign for the effectiveness of the calculations. The large dataset collected also uncovered structure–property relationships in the materials, as with the previous case study, as well as revealing upper bounds on the properties of the organic molecules. To select which of the 900 identified molecules to attempt to experimentally validate, a custom web tool was developed by Gómez‐Bombarelli et al. to allow humans to vote on which molecules to synthesize and fabricate into devices. The selected molecules were then produced, with the computational predictions agreeing well with the experimental performance. The discovered molecules had encouraging external quantum efficiencies (EQE) of up to 22%. Again, this study highlights the value of tiered computational‐experimental strategies that use high‐throughput computational screening to filter candidate libraries that are many orders of magnitude larger than anything that could feasibly be attempted synthetically, even with advances in automation and robotics.

### 3.9 Literature‐Mining to Discover Dye‐Sensitized Solar Cells (DSSCs)

DSSCs are a type of photovoltaic solar cells where the photoactive component of the solar cell is an organic dye. DSSCs have potential in wearable devices and textiles due to their flexibility, low cost, and scalable processing procedures. As with all‐organic materials, the large number of potential components, in this case light‐harvesting chromophores, is both a blessing and a curse—how does one effectively find optimal candidates when design of the materials with targeted properties is fundamentally not possible? Cooper et al. used an alternative approach to the manual collation of candidate libraries described in the previous two case studies.^[^ ^46^ ^]^ They used the ChemDataExtractor (CDE), previously developed by Swain and Cole for the automated extraction of chemical information from the literature, to data‐mine for potential dye candidates.^[^ ^45^ ^]^

**Figure 10:**
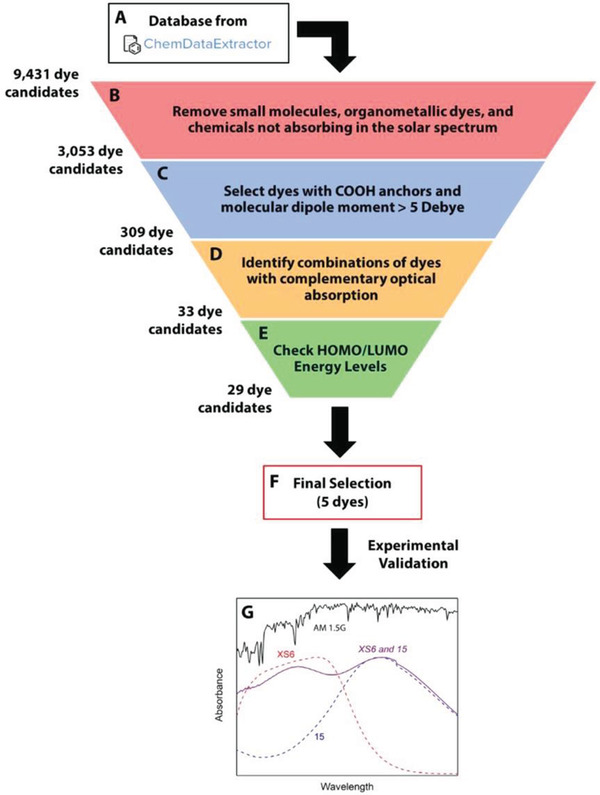
Data‐mining the literature for potential dye candidates for DSSCs; A) A starting database of 9431 dye candidates from the scientific literature was generated using the text‐mining tool, ChemDataExtractor. B) Initial screens remove small molecules, organometallic dyes, and chemicals not absorbing in the solar spectrum. C) Molecules lacking a carboxylic acid or reasonable dipole moment are removed. D) Optical properties of the dyes are assessed. E) HOMO and LUMO energy levels of each dye are checked using DFT to ensure proper integration into a DSSC. F) A final set of five dyes is selected for experimental verification. G) Experimental validation. Reproduced with permission.^[^ ^46^ ^]^ Copyright 2018, Wiley‐VCH.

CDE works to text‐mine chemical literature by automated extraction of chemical entities, and their associated reported properties, to autogenerate databases of known materials.^[^ ^45^ ^]^ The approach applies machine learning, including unsupervised learning, to tokenize text, based on training from a corpus of chemical literature. The software is extendible to training to text‐mine new properties of chemicals. Cooper et al. used CDE to create a database of 9431 dye molecules, including their chemical structure, molar extinction coefficients, and maximum absorption wavelengths. They conducted an initial screen of the library to remove dyes containing metals, small molecules, and those without absorbance in the solar spectrum, resulting in a filtered database of 3053 organic dyes (**Figure** **10**). Next, they filtered out dyes that did not contain a carboxylic acid group (to anchor it to the surface of titania in a DSSC device), and those with a small dipole moment, insufficient for intramolecular charge transfer upon photoexcitation. There were 309 molecules in the remaining subset.

In the following stage, Cooper et al. came up with a figure of merit for the candidate dyes that considered their likely performance based on their optical absorption properties, selecting 33 promising dyes. For these dyes, they calculated the HOMO and LUMO levels with DFT calculations, to confirm they were consistent with device integration with titania. From the remaining 29 candidates, 5 were selected for experimental validation based upon the ease of synthesis. The experimental device performance was promising, with high power conversion efficiencies, comparable to the industry standard, particularly when two of the five candidates were combined. This case study highlights the value of examining again the chemical literature, in an efficient, automated fashion, to uncover molecules with hidden, unrealized, capabilities.

### 3.10 Autonomous Exploration and Automated Synthesis of Metal–Organic Architectures

In the majority of the case studies discussed so far, a human has been involved in either the precursor selection or in narrowing down and selecting which computationally predicted materials to target. However, this can introduce bias and may reduce the chance of accessing truly novel and serendipitous discoveries. One approach is to screen and analyses every single possible variation for a particular materials class, but as highlighted in the other studies, this is not viable even with automated approaches. This could, however, be overcome by combining automated screening led by autonomous decision making, with a focus on finding new areas of reactivity.

Recently, Porwol et al. used such an approach to explore the area of coordination architectures.^[^ ^58^ ^]^ By combining closed‐loop automated flow screening with a search algorithm that was not trained with a dataset, the authors investigated the available chemical space for metal–organic supramolecular species formed from the self‐assembly of three chemical inputs (organic azides, aminoalkynes, and aldehydes) and two different coordination metals. In addition, the reaction conditions including the volume, stoichiometric ratio, reaction time, and temperature, were not predetermined or preset. Overall, even with this initially small selection of precursors, this therefore adds up to millions of possible reactions—screening all of these combinations is clearly not experimentally viable. The use of an algorithm to autonomously make decisions and search the chemical space was reliant on the initial choice of chemical inputs rather than a training set, and did not build a model of the space or optimize the reactivity during its exploration. Instead, the algorithm determined the degree of change from the chemical inputs, to the organic ligand, to the metal–organic assembly, using a combination of UV–vis measurements, mass spectrometry, and pH changes. Using this as an exploration factor, the algorithm then determined whether to continue investigating a similar region of chemical space as it had identified an area of high reactivity, or to search further away to find enhanced reactivity. Overall, this method autonomously discovered a range of ligands and metal–organic architectures in solution, and highlighted the benefits of using a closed‐loop autonomous system to actively investigate the reactivity space of a specific system. One current limitation is that the precursors were still selected by an experienced chemist, and while the ligand system targeted was unknown, it could be argued that the target assemblies were based on prior chemical knowledge of other known ligand binding motifs. However, this is still a significant advance in the area of integrating computation and automated screening in a closed feedback loop for the discovery of materials, without direct human involvement during the screening process.

## 4 Future Outlook

The future is bright for integrated computational–experimental organic materials discovery. We have an increasingly computer literate generation of materials chemists, with many experimental chemists proficient in coding in at least one language, commonly Python. This combines with a move toward more open‐source software, the availability of which has contributed to the explosion of studies in AI, and the increasing move toward the creation of open‐source materials databases and electronic laboratory notebooks. The use of automation to streamline and reduce the experimental timescale has also seen increased interest, although arguably the uptake is slow due to the typically associated high cost of such systems. As the development of low‐cost and open‐source automation becomes more widespread, and when combined with increased computational proficiency, these approaches and systems will hopefully become more common place in research labs and be applied to different research programmes to streamline and accelerate discovery. Further to this, there is also a need for accessible application programming interfaces (API) in laboratory equipment to allow device modification and facilitate data collection, or for the integration of open‐source and repurposed robotic arms and sensors with standard equipment to overcome the need to modify certain devices. For example, in recent work by Burger et al., a commercially available mobile robotic arm was programmed to interact with typical equipment found in a laboratory.^[^ ^59^ ^]^ There are also exciting developments in terms of integrating experiment and computation, allowing human interaction during a workflow, such as the ChemVox software of Martínez and co‐workers which can perform computations based on natural language processing from a voice command^[^ ^60^ ^]^ or via virtual reality and calculations responding to human movements.^[^ ^61^ ^]^

We can hope AI algorithms can provide new ways to explore the chemical space of precursors, so that we can uncover “wild cards” that would not otherwise be suggested by humans alone. In this area, a particularly influential paper by Gómez‐Bombarelli et al. made use of a deep neural network (DNN) as part of a variational autoencoder (VAE).^[^ ^62^ ^]^ In this case, the approach was applied to molecular properties such as solubility, rather than material properties, but it will be interesting to see how successfully it can be applied to organic materials. The DNN encodes the molecular SMILES‐based representations into a latent space, then the chemical space can be explored, before converting, via a decoder, back to the molecular representation. As property predictions can be performed in the latent space, this opens up the possibility for inverse design. One potential issue is that many of the new molecules may not be synthetically accessible, so workflows will need to consider this point. So while, in the above discussion of accelerated property prediction, these were all examples of forward prediction, these types of algorithms can help to open up the possibility for truly performing “inverse design” of materials based on designing a material with optimal properties. The challenge of multiobjective optimization of multiple properties remains.

Improvements in the automation of precursor synthesis can help us to reduce that bottleneck in the materials discovery process. This would facilitate a large number of materials being experimentally tested. An interesting development in the use of AI in chemistry are algorithms that, for a given organic molecule, can perform a “computational retrosynthesis” to predict the most effective, and even cheapest, synthesis route to that molecule.^[^ ^63^, ^64^, ^65^ ^]^ These approaches typically use deep learning or reinforcement learning and have typically been trained on known reaction data, but have more recently been trained only based on known reaction classes. While currently focused in organic chemistry, such algorithms also hold promise in helping guide materials discovery, by aiding in the identification of which promising molecular building blocks for materials are synthetically reachable. This will hopefully help embolden experimental chemists to make precursor choices that are less similar to previously reported molecules and thus help us explore more chemical space beyond small, unimaginative starting libraries.

The next stage, material synthesis, can hopefully be guided in the future by predictions of the reaction outcomes, or, even better, for the required reaction and processing conditions to be predicted a priori, saving vast amounts of time exploring the reaction phase space experimentally. There are recent reports of reaction condition prediction for small molecule organic reactions,^[^ ^66^ ^]^ as well as for zeolite synthesis,^[^ ^67^ ^]^ and, as discussed in the case studies, MOF synthesis. These studies have used literature data, or collected their own data. The diversity and scale of reaction data for organic material synthesis is not currently available, but this can now be remedied for specific reaction classes by the high‐throughput collection of data through automated synthesis. Again, it is critical that data for both successful and failed reactions are collected and shared.

CSP for solid‐state molecular packing of materials has the potential to be vastly accelerated by ML, extending the scale at which it can be applied to materials discovery, and hopefully improving the accuracy of property predictions. Of particular promise is the use of ML targeted at the bottleneck of the CSP process, the accurate energetic ranking of the hypothetical polymorphs. One recent example is in the use of ML‐based fragment energies, shown to improve the relative energies of hypothetical polymorphs.^[^ ^68^ ^]^ Accelerated CSP, combined with ML‐based property predictions would massively accelerate the computational screening of organic materials. ML‐based property prediction, already a very popular field, will be increasingly used and successful as new algorithms become available, and as the quantity of data openly available increases.

Finally, the use of closed feedback loops and autonomous workflows combining both computational prediction and high‐throughput experiment is already starting to emerge as a powerful tool for the discovery of materials, particularly in the area of optimization. For example, Langer et al. recently reported the autonomous optimization of multicomponent polymer blends for organic photovoltaics by combining an automated platform that can rapidly fabricate films, with a Bayesian optimization.^[^ ^69^ ^]^ Burger et al. took this approach even further, by combining a mobile robot and a Bayesian search algorithm to autonomously search for photocatalysts with improved hydrogen production performance.^[^ ^59^ ^]^ Overall, this area of research will open up the next generation of self‐driving autonomous laboratories, and hopefully lead to more imaginative and accelerated leaps in the design and discovery of new materials.

## 5 Conclusions

We have discussed the state‐of‐the‐art in integrating computational and experimental materials discovery programmes for organic materials with application in guest storage, separations, optoelectronics, and (photo)catalysis. Integrated workflows can use computation to perform molecular design, and to screen vast libraries of possible materials, many orders of magnitude larger than anything that can be experimentally synthesized, even with automation. The likely products and structures of the materials formed are now beginning to be able to be predicted, so that we can target material synthesis toward materials that are synthetically achievable. Artificial intelligence will continue to impact the field, and has already shown much promise in accelerating property predictions, something that will only increase as larger databases of materials and their properties become available. High‐throughput automation can be used to accelerate the experimental timescale for screening for the optimal conditions required for a particular materials synthesis, or to screen for a range of materials. While not currently widely utilized, as more automated platforms become available and low‐cost systems developed, the use of automation will surely be adopted by other researchers. For the future, automation and artificial intelligence will continue to accelerate both experimental and computational programmes, especially when they are integrated to feed into each other.

## Conflict of Interest

The authors declare no conflict of interest.
